# Challenges in the Management of Glioblastoma in a Developing Country: Experience From the Radiotherapy Oncology Department in Marrakech, Morocco

**DOI:** 10.7759/cureus.46258

**Published:** 2023-09-30

**Authors:** Mouna Darfaoui, Yassir Tahiri, Abdelhamid Elomrani, Mouna Khouchani

**Affiliations:** 1 Radiation Oncology, Mohammed VI University Hospital, Marrakech, MAR

**Keywords:** management challenges, healthcare inequality, therapeutic advances, developing countries, glioblastoma

## Abstract

Managing glioblastoma (GBM) is challenging even for the most experienced centers in high-income countries due to its infiltrative nature, its unique tumor and immune microenvironment, and the negative effect of the blood-brain barrier on the penetration of systemic therapies. In developing countries, the difficulties are even greater, mostly in relation to the lack of adequate medical infrastructure and resources. This paper highlights the disparities in GBM management between developed and developing countries. Throughout this retrospective study conducted at the Radiation Oncology Department of Mohammed VI University Hospital in Marrakech, Morocco, we investigated the management outcomes of 48 GBM patients diagnosed between 2016 and 2021. Results showed a male predominance (65%) and a mean age of 53 years. Gross total resection was achieved in 16% of the patients and subtotal resection in 80%. Adjuvant radiotherapy was pursued, with a prescribed dose of 60 Gray in 30 fractions of 2 Gray for most patients. Concurrent temozolomide was administered to 32 patients (66.6%) with favorable tolerance. However, disease progression occurred in all cases, with a median time to progression of five months and a median survival of eight months. In conclusion, a comprehensive awareness of our limitations empowers us to implement measures that secure impartial access to standard-of-care treatments for every patient in Morocco, ultimately elevating the effectiveness of therapeutic outcomes.

## Introduction

Glioblastoma (GBM) is the most common malignant primary tumor of the brain, accounting for 14.5% of all central nervous system tumors and 48.6% of malignant central nervous system tumors [1].

Despite the standard-of-care multimodal therapy, including maximal safe resection, radiotherapy, and chemotherapy, the prognosis remains almost universally fatal with a mean overall survival of 14 to 20 months [1-2].

In 2021, the World Health Organization published the fifth edition of the Classification of Central Nervous System Tumors. The major changes were driven by IDH mutation status and included the restriction of the diagnosis of GBM to tumors that are IDH wild type; tumors previously diagnosed as IDH-mutated GBMs were reclassified as astrocytoma IDH mutated, grade 4 [3].

In recent years, advancements in molecular profiling and targeted therapies have shown promise in addressing some of the complexities associated with GBM. Personalized treatment approaches, guided by the molecular characteristics of each patient's tumor, are emerging as potential avenues to improve outcomes [4].

The management of GBM in low- to medium-resource countries is impeded by escalating economic constraints, insufficient clinical testing capabilities, and limited access to some therapeutic drugs [5].

This study investigates the challenges faced in managing GBM within a developing country, drawing insights from the Radiation Oncology Department of Mohammed VI University Hospital in Marrakech, Morocco. It explores constraints arising from resource scarcity, economic limitations, constrained clinical testing, and limited availability of essential therapeutic drugs. The article contributes to the discourse on optimizing GBM management strategies in low-resource settings.

## Materials and methods

Study design and population

This retrospective cohort study was conducted at the Radiation Oncology Department of Mohammed VI University Hospital in Marrakech, Morocco. The study included a total of 48 patients diagnosed with GBM between the years 2016 and 2021.

Data collection

Patient data were collected from electronic medical records and treatment planning systems. Information included demographic details, clinical history, diagnostic imaging, treatment regimens, and follow-up outcomes.

Inclusion and exclusion criteria

Inclusion criteria encompassed patients with a confirmed diagnosis of GBM IDH1 wild type based on histopathological examination. Patients with incomplete records were excluded.

Ethical considerations

The study was approved by the local ethical committee of Mohammed VI University Hospital (FCR-CS-09/2023-0001). The anonymity of patients was ensured and respected throughout the study.

## Results

The baseline patient, tumor, and treatment characteristics were summarized in Table 1. The time to diagnosis varied from one to 12 months, with a median time of 4.2 months. The main symptoms observed were motor deficits in 52% of cases, intracranial hypertension in 48% of cases, and higher function deficits in 16.6% of cases. According to the Karnofsky status assessment, 33 patients scored 80%, nine scored 60%, and six scored 60%.

**Table 1:** Patients' demographic characteristics GTR: guided tissue regeneration, STR: selective dorsal rhizotomy, RT: radiotherapy, TMZ: temozolomide

		Number of patients	Percent %
Gender	Male	31	65
	Female	17	35
Age	20-30	3	6.2
	31-40	5	10.4
	41-50	12	25
	51-60	12	25
	>60	16	33.3
Location	Parietal lobe	31	65
	Temporal lobe	22	46
	Frontal lobe	12	25
	Occipital lobe	3	6.2
	Thalamus	1	2
Size	<30 mm	7	14.5
	30 mm	41	85.5
Type of surgery	GTR	8	16.7
	STR	38	79.1
	Biopsy	2	4.2
Adjuvant treatment	Stupp protocol	15	31.2
	RT alone	5	10.4
	Concomitant RT-TMZ	17	35.4
	Sequential RT-TMZ	11	22.9

**Figure 1:**
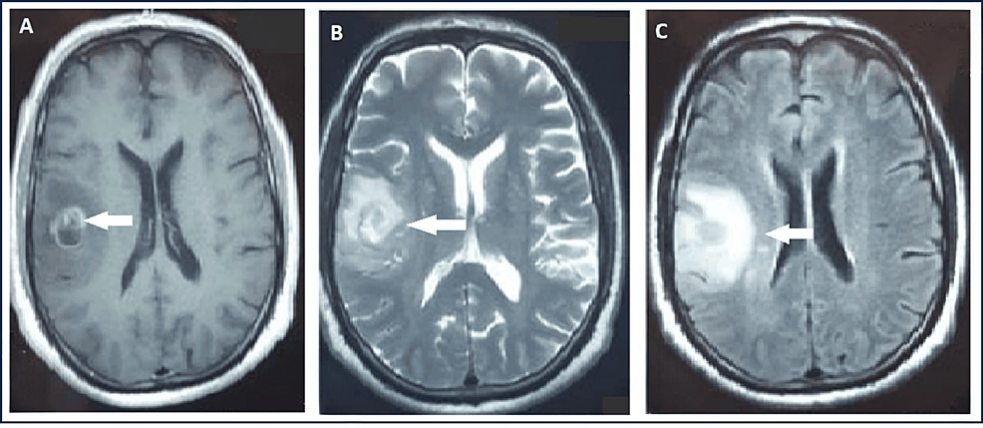
MRI aspect of a high-grade GBM in a contrast-enhanced T1-weighted axial image (A) with heterogeneous irregular peripheral enhancement associated with the right parietal lobe mass and central non-enhancing area, consistent with necrosis and associated mass effect on the right lateral ventricle, T2 (B) and flair sequence (C) showing a heterogeneous mass centered in the right parietal lobe with surrounding infiltrating signal abnormality Arrows highlight the lesion on the different sequences.

Analysis of MRI results revealed the distribution of tumor locations within our study group. The most prevalent locations were the parietal and temporo-parietal regions, accounting for 27% and 25% of cases, respectively. Following this, tumors were found in the temporal (14.5%), front-parietal (12.5%), and frontal (12.5%) regions. Less frequent tumor sites included occipital (6%) and thalamus (2%) regions. Tumor dimensions were assessed along the greater axis. The recorded sizes ranged from 19 mm to 72 mm, with a median size of 44 mm (Figure 1).

The cornerstone of our approach was surgical intervention, undertaken for all patients within our series. Gross total resection was achieved in eight patients (16%). In 38 cases (80%), a subtotal resection was performed, and in two cases (4%), a stereotaxic biopsy was chosen and performed. Post-operative MRI, performed in the first 72 hours, revealed a reduction in tumor size for almost all our patients, with a median size of 29.1 mm and a range spanning from 0 to 68 mm.

All our patients underwent adjuvant radiotherapy, and the technique used was a three-dimensional conformational radiotherapy. We assessed the time gap between surgery and radiotherapy, which varied from 3 to 21 weeks, with a median delay of 11 weeks. The prescribed dose was 60 Gray in 30 fractions for 46 patients and 40 Gray in 15 fractions for two patients. Unfortunately, six patients were unable to complete the full course of radiotherapy due to disease progression and deteriorating health conditions.

In terms of concurrent temozolomide, it was administered to 32 patients with overall favorable tolerance. In the adjuvant setting, temozolomide was completed for six cycles in 17 patients, with a median of three cycles per patient. The Stupp protocol [2] was successfully completed in 15 patients, while radiotherapy was delivered as the only treatment in five patients.

At a median follow-up of seven months (range, 1-29 months), all the patients experienced a progression of their disease. This progression happened within a range of 0 to 22 months after completing radiotherapy, with a median time to progression of five months. Survival was determined by time from diagnosis to death. This span ranged from 2 to 27 months, with a median survival duration of eight months.

As salvage treatment after progression in our cohort, three patients received targeted therapy with bevacizumab, five patients were eligible for reoperation, and no one underwent salvage stereotactic radio-surgery.

## Discussion

Throughout the literature, the incidence of GBM shows locational variability and ranges from 0.76 in Iran to 4.8 in Croatia [6]. In Morocco, we lack information regarding the incidence of GBM. However, based on the cancer registry of Greater Casablanca, the incidence of central nervous system tumors is reported to be 2.1 per 100,000 inhabitants, with GBM accounting for 40% of these cases [7].

Numerous investigations affirm the substantial influence of age on GBM incidence, with a predominant majority of cases emerging in individuals aged over 40. In concordance with a Jordanian cohort, our study demonstrates that more than 60% of patients were below 50 years old [8]. This deviates from the findings in an analysis of the Surveillance, Epidemiology, and End Results (SEER) database, where 64% of patients were 54 years or older [1]. Increasing age is known as an adverse prognostic factor for survival with GBM. This has been uniformly reported since the first therapeutic studies from the 1970s to actual large multicenter-based data [1,8].

Molecular testing has emerged as a critical component in the management of GBM [4]. However, implementing molecular testing in a resource-constrained setting like Morocco presents significant challenges. The availability of specialized laboratories and cutting-edge equipment is often limited. Additionally, the cost associated with molecular testing can strain healthcare budgets, potentially limiting its accessibility for many patients. Despite these limits, recognizing the potential benefits of molecular testing for guiding diagnosis, therapeutic strategy, and predicting treatment responses emphasizes the need for concerted efforts to enhance infrastructure, expertise, and affordability in order to effectively integrate this essential tool into the management of GBM within the Moroccan healthcare system.

All our patients received adjuvant radiotherapy; the challenge was to ensure its prompt delivery within acceptable delays. The analysis of the National Cancer Database suggests that for patients with newly diagnosed GBM, a radiotherapy delay of four to eight weeks following resection is associated with better overall survival. The impact of time between surgery and RT was dependent on the extent of resection. Particularly in patients with gross total resection, delays of longer than eight weeks were associated with worse survival, making them comparable to patients who receive a subtotal resection [9].

In 2005, Stupp et al. [2] reported significantly longer survival in patients treated with temozolomide in addition to radiotherapy which completely revolutionized the treatment of GBM. Post-operative radiotherapy with concomitant and adjuvant temozolomide is now the standard treatment for GBM [2]. In our study, access to this treatment displayed inequalities among patients, based on their socio-economic circumstances, thereby compromising their overall survival.

On average, the survival rate for GBM is relatively low. The median survival time is around 15 months with the current standard treatments. A small percentage of patients can survive for more than five years or even longer. The five-year survival rate is often less than 10%, but it can vary based on many prognostic factors, especially the type of surgery, delay to radiotherapy, and adjunction of chemotherapy [10].

The present survival rates observed in our cohort are below those recorded by Stupp et al. [10]. This contrast is anticipated to be even more pronounced in the era of personalized treatment, where the emerging prominence of targeted therapies and immunotherapy is significantly influencing the management of recurrent GBM [4,11,12]. However, it's worth noting that while targeted therapies and immunotherapy offer promising benefits, their expensive nature could potentially exacerbate the disparities in accessing standard-of-care treatment for GBM patients.

In Morocco, healthcare services are delivered through a network of public, university, and private hospitals. Public hospitals, managed by the Ministry of Health, ensure accessible primary care coverage. University hospitals specialize in advanced tertiary care, while private hospitals operate on a fee-based model.

Additionally, the Foundation Lalla Salma, a non-lucrative association specializing in cancer care in Morocco, plays a significant role in enhancing healthcare services and access to expensive medications for cancer patients in Morocco. Their efforts contribute to a more robust and comprehensive healthcare landscape, offering a promising avenue for addressing the challenges faced by GBM patients and others in need of specialized care. Lately, the generalization of basic health insurance coverage in Morocco since December 2022 has brought about notable improvements, yet there are still disparities in care.

Despite these efforts, challenges persist in extending comprehensive quality care for GBM patients. These challenges include a lack of national or regional cancer registries, non-mandatory multidisciplinary tumor board meetings, and an uneven distribution of care providers across the nation. This therapeutic variability aligns with patterns often observed in developing nations, where resource limitations further impact countries with lower income levels, like Morocco.

Another challenging condition for low- and middle-income countries is the limited interest, capacity, and infrastructure for brain cancer research. Recently, some developing countries, including Morocco, have witnessed some improvements in neuro-oncological research. In addition, the increasing burden of GBM and the associated increased demands for therapeutic drugs urge pharmaceutical companies to sponsor brain cancer research in resource-limited countries.

All these difficulties result in lower survival in our cohort compared to those in the literature.

The limitations of this study include mainly its relatively small sample size of 48 patients, rendering it less representative of the entire spectrum of GBM cases. Being a single-center, retrospective study introduces potential biases and may not capture the diversity of patient experiences. Additionally, the relatively short median follow-up period of seven months limits the assessment of long-term survival outcomes.

## Conclusions

Our retrospective cohort study of GBM management in Morocco reveals the challenges and outcomes faced by patients in this context. The study underscores the importance of equitable access to advanced treatments such as radiotherapy and concurrent temozolomide in resource-limited settings. Understanding these complexities paves the way for informed decision-making and targeted interventions, aiming to improve therapeutic results and ultimately enhance patients' quality of life. By acknowledging our shortcomings and working collaboratively to bridge the gaps, we can strive to ensure comprehensive and effective GBM care for all patients in Morocco, thereby contributing to the global efforts in combating this challenging malignancy.
